# Synthesis and Molecular-cellular Mechanistic Study of Pyridine Derivative of Dacarbazine

**Published:** 2013

**Authors:** Marzieh Amirmostofian, Jalal Pourahmad Jaktaji, Zohreh Soleimani, Kimia Tabib, Farahnaz Tanbakosazan, Mirdavood Omrani, Farzad Kobarfard

**Affiliations:** a *Department of Medicinal Chemistry, School of Pharmacy, Shahid Beheshti University of Medical Sciences, Tehran, Iran.*; b *Department of Pharmacology and Toxicology, School of Pharmacy, Shahid Beheshti University of Medical Sciences, Tehran, Iran.*; c *Department of Genetics, School of Medicine, Shahid Beheshti University of Medical Sciences, Tehran, Iran. *

**Keywords:** Dacarbazine, Pyridine, Cytotoxicity, Oxidative stress, Lysosome, Mitochondrial Membrane potential

## Abstract

Dacarbazine is an antitumor prodrug which is used for the treatment of malignant metastatic melanoma and Hodgkin’s disease. It requires initial activation in liver through an N-demethylationreaction. The active metabolite prevents the progress of disease via alkylation of guanine bases in DNA strands. In order to investigate the importance of imidazole ring and its dynamictautomerization in anticancer activity of dacarbazine, a pyridine analog of this drug was synthesized and the cytotoxic activity and cellular-molecular mechanisms of action for this compound were compared with those of dacarbazine. EC50 values for dacarbazine and the pyridine analog were found to be 56 μM and 33 μM respectively. Both dacarbazine and the pyridine analog resulted in formation of reactive oxygen species (ROS) upon their addition to the isolated rat hepatocytes. They also decreased the mitochondrial membrane potential and causedlysosomal membrane rupture. Cytotoxicity was prevented by ROS scavengers and antioxidants. Cytotoxicity wasalso prevented by CYP_450 _inhibitors, lysosomalinactivators and MPT (Mitochondrial Permeability Transition Pore) blockers.

## Introduction

**Figure 1:**
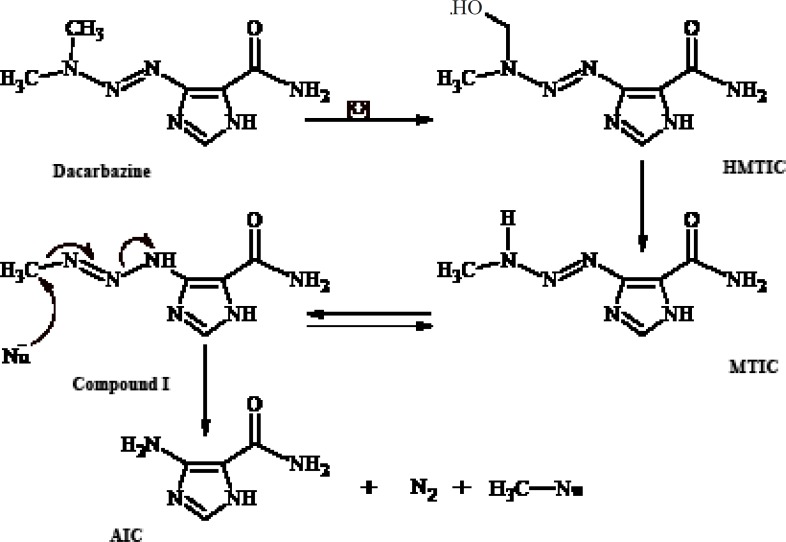
*In-vivo *metabolism of dacarbazine and alkylation of nucleophiles (Nu) by dacarbazine metabolite I

Dacarbazine (Figure 1) is an antitumor prodrugused for the treatment of malignant metastatic melanoma and Hodgkin’s disease (1- 3). Ithas an imidazole ring in its structure and is structurally a 1-aryl-3,3-dimethyltriazene thatundergoes *in-vivo *metabolic *N*-demethylation (4, 5) to yield ultimately 5-aminoimidazole-4- carboxamide(AIC) (Figure 1). The metabolic precursor of 5-aminoimidazole-4-carboxamide (AIC) is 5-(3-methyltriazene-1-yl)imidazole- 4-carboxamide (MTIC)(3, 4, 6-8) thatis a short-lived species. MTIC is believed to be the metabolitethroughwhich dacarbazine exerts its antineoplastic alkylation effect. MTIC undergoes tautomerization to a methylating species (compound I), thatreacts with nucleophiles such as DNA guanine bases and concomitantly generates 5-aminoimidazole-4-carboxamide (AIC) (9). 

**Figure 2:**
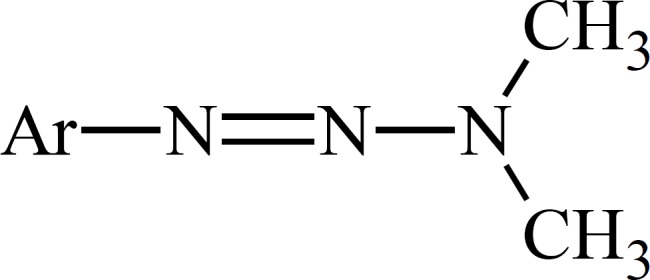
Title missing

The role of metabolism in the mode of cytotoxic action of dacarbazine has been deduced from the experiments on dimethyltriazenesthatpossess the substituted phenyl group instead of the imidazole moiety on N1 (Figure 2).

Metabolism of such 1-phenyl-3,3-dimethyltriazenes generates cytotoxic monomethyltriazenes which act just like intermediate I (Figure 1) in the alkylation of nucleophile groups in the tissue.The immediate metabolic precursor of MTIC, 5-[3-(hydroxymethyl)-3-methyltriazene-1-yl]imidazole-4-carboxamide (HMTIC) (10) has been characterized as a urinary metabolite of dacarbazine and this compound is believed to act as a transported form of MTIC, which is the suggested antineoplastic species derived from dacarbazine. 

**Figure 3:**
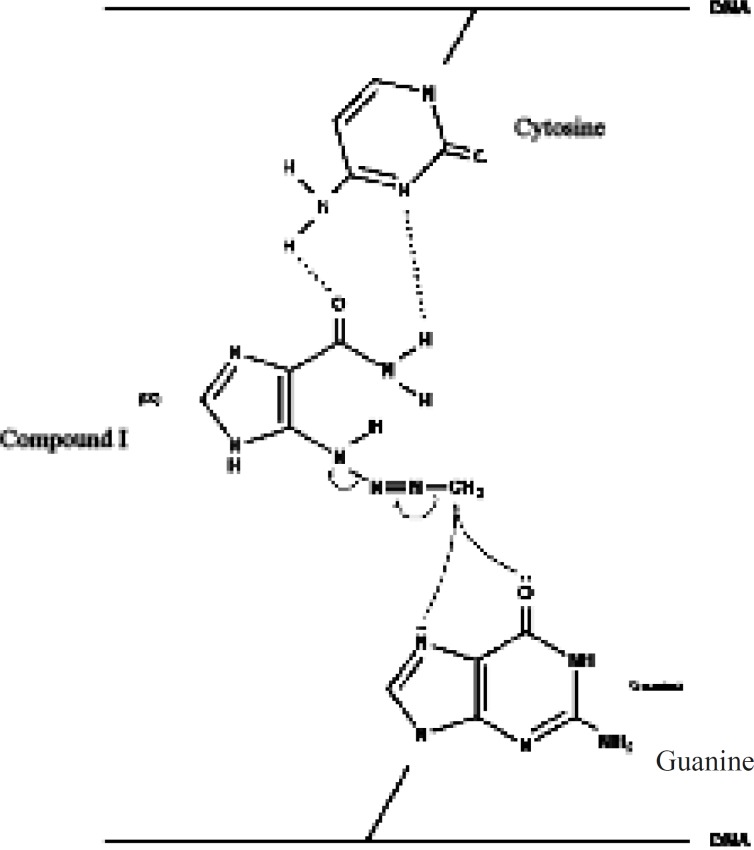
Title missing

Based onthe rather selective methylation of guanine bases by dacarbazine, Lowe *et al*. (11) postulated that the carboxamide group in dacarbazine plays a recognition role in finding guanine rich moieties in DNA strands. Carboxamide group in one of its conformationsforms hydrogen bonds with cytosine similar to that of guanine. The alkylating methyltriazene group of Compound I is therefore placed in the proximity of guanine and alkylation will take place at guanine O-6 and *N*-7 moiety (Figure 3) (3, 6, 12-15).

**Figure 4:**
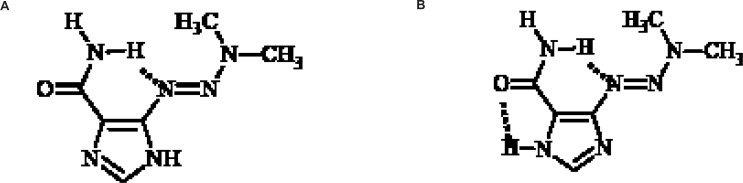
Two tautomeric forms of dacarbazine. a: NaNO_2_ , HCl, b: Suspension of Na_2_SO_4_ and 40% solution of dimethylamine in H_2_O

The imidazole ring could exist in two tautomeric forms and X-ray crystal structure studies of dacarbazine shows that this compound exists in two tautomeric forms a and b as shown in Figure 4.

Tautomeric form b is more stable due to the two intramolecular hydrogen bonds. The role of dynamic tautomerization of imidazole ring in dacarbazine alkylating activity has not been well documented yet (16). In this study a dacarbazine analog wassynthesized in which the imidazole ringwasreplaced by pyridine ring which could be considered as the non-tautomerizable form of imidazole ring. Evaluation and comparison of cytotoxic effect of pyridine congener and dacarbazinerevealed the role of imidazole tautomerization in dacarbazinecytotoxic activity.

**Figure 5:**
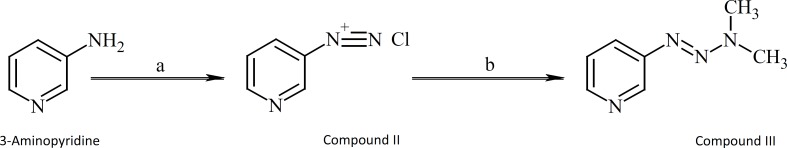
Synthesis of compound III

3-[3,3-Dimethyl-1-triazenyl]pyridine (compound III) was synthesized as shown in Figure 5. 

## Experimental

*Chemicals*

5-(3,3-dimethyl-1-triazeno)-imidazole- 4-carboxamide (Dacarbazine, DTIC) was purchased from Fehlandtstrabe 3.D-20354 (Hamburg, Germany). All the other chemicals were ofsynthesis grade and purchased from Merck (Darmstadt, Germany). Collagenase (from *Clostridium histolyticum*), bovine serum albumin (BSA), Hepes, trypan blue, d mannitol, dimethyl sulfoxide, catalase, superoxide dismutase (SOD), cyclosporine, butylatedhydroxyltoluene (BHT), chloroquinediphosphate, methylamine HCl, ethylene glycol bis (p aminoethyl ether) N,NN’,N’ tetra acetic acid (EGTA) and heparin were obtained from Sigma (Taufkirchen, Germany). Carnitine was obtained from ICN Biomedicals (St. Thuringen, Eschwege, Germany). Acridine orange and dichlorofluorescindiacetate were purchased from Molecular Probes (Eugene, OR, USA). Rhodamine 123 was obtained from Aldrich Chemical Company (Milwaukee, WI, USA). Desferoxamine was a gift from CibaGeigy Canada Ltd. (Toronto, ON, Canada). All chemicals were of the highest commercial grade available. ″Sigma’s caspase3 assay kit (CASP-3-C)″ was purchased from Sigma-Aldrich (Taufkirchen, Germany).

*Chemistry*

A solution of sodium nitrite (0.02 mol, 1.38 g) in 3.5 mL of water was added dropwise to a solution of 3-aminopyridine (0.02 mol, 1.88 g) in concentrated HCl (8 mL) and water (5 mL) in an ice bath while the temperature was kept at 0 °C. The mixture was stirred for further15 minutes after completion of addition and then a solution of urea in water (0.1 g in 0.3 mL H_2_O) was added and the mixture stirred for 20 min. The mixture was then added to a mixture of sodium carbonate (7.42 g in 25 mL water) and 40% dimethylamin solution (6 mL), forming a red mixture. The mixture was then filtered and extracted by ethylactate (60 mL). The organic phase thus obtained was evaporated under vacuum after drying over anhydrous sodium sulfate. The crude product was further purified on a silicagel plate (60 GF 254) using ethylacetate:chloroform (1:1) as the eluent solvent. Yield = 61%. IR (KBr): λ 2900, 1435, 1410, 1390, 1375, 1330, 1200, 1080, 800, 1H-NMR(CDCl3) δ: 8.7 (1H;d,4J=2.1HZ;H-2), 8.3 (1H;d,3J=4.7HZ;H-6), 7.7 (1H;dt,3J=8.1HZ, 4J=1.7HZ;H-4), 7.25 (1H;dd,3J=8.1HZ, 3J=4.7HZ;H-5), 3.5 & 3.25 (6H;two broad singlets;CH_3_), Mass (EI), M/Z (%): 150 (82), 121 (40), 106 (86), 92 (28), 78 (100).

*Animals*

Male Sprague-Dawley rats (280-300 g) fed a standard chow diet and given water ad libitum, were used in all the experiments. All the experiments were conducted according to the ethical standards and protocols approved by the Committee of Animal Experimentation of ShahidBeheshti University of Medical Sciences, Tehran, IR Iran.

*Isolation and incubation of hepatocytes*

Hepatocytes were obtained by collagenase perfusion of the liver as described by Pourahmad and O’Brien (17). Approximately 85-90% of the hepatocytes excluded trypan blue. Cells were suspended at a density of 106cells/mL in round bottomed flasks rotating in a water bath maintained at 37C in Krebs-Henseleit buffer (pH 7.4), supplemented with 12.5 mMHepes under an atmosphere of 10% O_2_, 85% N_2_, 5% CO_2_. Each flask contained 10 mL of hepatocyte suspension. Hepatocytes were preincubated for 30 min prior to the addition of chemicals. Stock solutions of all chemicals (×100 concentrated for the water solutions or ×1000 concentrated for the methanolic solutions) were prepared fresh prior to use. To avoid either nontoxic or very toxic conditions in this study we used EC50_2h_ concentration for DTIC and Compound III in the isolated hepatocytes (56 μM and 33 μM respectively). The EC50 of a chemical inACMS technique (ACMS: Accelerated Cytotoxicity Mechanism Screening) (with the total 3 h incubation period), is defined as the concentration which decreases the hepatocyte viability down to 50% following the 2 h of incubation (18). In order to determine this value for the investigated compound, dose-response curves were plotted and then EC50 was determined based on a regression plot of three different concentrations (data and curves not shown) (19). For the chemicals soluble in water, we added 100 μL sample of its concentrated stock solution (×100 concentrated) to one rotating flask containing 10 mL hepatocyte suspension. For the chemicals soluble in methanol we prepared methanolic stock solutions (×1000 concentrated), and to achieve the required concentration in the hepatocytes, we added 10 μL samples of the stock solution to the 10 mL cell suspension. Ten microlitres of methanol did not affect the hepatocyte viability after 4 h incubation (data not shown). All the inhibitors were preincubated 30 min prior to DTIC and Compound III addition.

*Cell viability*

The viability of isolated hepatocytes was assessed from the intactness of the plasma membrane as determined by the trypan blue (0.2% w/v) exclusion test (20). Aliquots of the hepatocyte incubate were taken at different time points during the 3 h incubation period. At least 80-90% of the control cells were still viable after 3 h.

*Determination of reactive oxygen species (ROS)*

To determine the rate of hepatocyte ROS generation, dichlorofluorescindiacetate (DCFH-DA) was added to the incubatedhepatocytesas it penetrates hepatocytes and becomes hydrolyzed to non-fluorescent dichlorofluorescin (DCFH). The latter then reacts with ROS to form the highly fluorescent dichlorofluorescein (DCF), which effluxes the cell. Hepatocytes (1 106 cells/mL) were suspended in 10ml modified Hank’s balanced salt solution (HBS), adjusted to pH 7.4 with 10 mM HEPES (2-hydroxyethyl)-1-piperazine –ethansulfonic acid-HBSH) and were incubated with DTIC and Compound III at 37°C for 30 min. After centrifugation (50 × g. 1 min), the cells were re-suspended in HBS adjusted to pH 7.4 with 50 mM Tris-HCl and loaded with dichlorofluorescin by incubating with 1.6μL dichlorofluorescindiacetate for 2 min at 37°C. The fluorescence intensity of the ROS product was measured using a Shimadzu RF5000U fluorescence spectrophotometer. Excitation and emission wavelengths were 500 nm and 520 nm, respectively. The results were expressed as fluorescent intensity per 106 cells (20, 21).

*Lysosomal membrane stability assay*

Hepatocyte lysosomal membrane stability was determined from the redistribution of the fluorescent dye, acridine orange (22). Aliquots of the cell suspension (0.5 mL) that were previously stained with acridine orange 5 μM, were separated from the incubation medium by 1min centrifugation at 1000 rpm (rotations per min). The cell pellet was then re-suspended in 2 mL of fresh incubation medium. This washing process was carried out for two times to remove the fluorescent dye from the media. Acridine orange redistribution in the cell suspension was then measured fluorimetrically using theShimadzu RF5000U fluorescence spectrophotometer set at 470 nm excitation and 540 nm emission wavelengths.

*Mitochondrial membrane potential assay*

Mitochondrial uptake of the cationic fluorescent dye, Rhodamine 123 (1.5 μM), has been used for the estimation of mitochondrial membrane potential (23). Aliquots of the cell suspension (0.5 mL) were separated from the incubation medium by centrifugation at 1000 rpm (rotations per minute) for 1 min. The cell pellet was then re-suspended in 2 mL of fresh incubation medium containing 1.5 μM Rhodamine 123, and incubated at 37 °C in a thermostatic bath for 10 min with gentle shaking. Hepatocytes were then separated by centrifugation and the amount of Rhodamine 123 remaining in the incubation medium was measured fluorimeterically using a Shimadzu RF5000U fluorescence spectrophotometer set at 490 nm excitation and 530 nm emission wavelengths. The capacity of mitochondria to take up the Rhodamine 123 was calculated as the difference (between control and treated cells) in Rhodamine 123 fluorescence. Our data were shown as the percentage of mitochondrial membrane potential collapse (%ΔΨm) in all treated (test) hepatocyte groups (23, 24).

*Determination of caspase-3 activity*

Caspase-3 activity was determined by using the ″Sigma’s caspase-3 assay kit (CASP-3-C)″ (Sigma-Aldrich, Taufkirchen, Germany). This measurement was performed based on the hydrolysis of Ac- DEVD-pNA peptide substrate by caspase-3. The released moiety (p-nitroaniline) has a high absorbance at 405 nm (25). 

$\mathrm{AC}-\mathrm{DEVD}-p\mathrm{NA}\vec{\mathrm{Caspase}-3}\mathrm{AC}-\mathrm{DEVD}+p\mathrm{NA}$

The concentration of the p-nitroaniline released from the substrate wascalculated from the absorbance values at 405 nm or from a calibration curve prepared with defined p-nitroaniline solutions. The activity of caspase-3 was obtained by pNA concentration (μM) using the following equation:

Caspase-3 activity, μM pNA / min / mL =μM pNA × d t × v

(t: time, v: volume of solution, d: dilution factor)

*Statistical analysis*

Levene´s test was used to check the homogeneity of variances. Data were analyzed using one-way analysis of variance (ANOVA) followed by Tukey Post-test. Results represent the mean ± standard deviation of the mean (S.D.) of triplicate samples. The minimal level of significance chosen was p ≤ 0.05.

## Results and Discussion

Using accelerated cytotoxicity mechanism screening (ACMS) technique, EC_50_ values were calculated as 56 μm for dacarbazine and 33 μm for compound III. These values indicate that pyridine derivative of dacarbazine (compound III) is almost two times more potent than dacarbazine. In order to investigate the molecular-cellular mechanism of cytotoxicity for compound III and dacarbazine, the effect of these compounds on hepatocyte cell death was evaluated in the presence of a wide variation of antioxidants (catalase, superoxide dismutase, *etc*.), ROS scavengers (mannitol, dimethylsulfoxide, *etc*.), a ferric chelator (desferoxamine), a CYP2E1 inhibitor (phenylimidazole), P_450_ reductase inhibitor (diphenyliodonium chloride - DPI), endocytosis inhibitors (chloroquineand methylamine) and mitochondrial permeability transitionpore inhibitors (cyclosporin and carnitine).In order to further investigate the mechanistic similarities between the cytotoxic activity of compound III and dacarbazine, the effect of these compounds on reactive oxygen species (ROS) formation, liposomal membrane leakiness and decrease in mitochondrial membrane potential were determinedby the measurement of the intensity of absorbance of fluorescence dyes with fluorescence spectrophotometer.

When hepatocytes were incubated with 56 μm of dacarbazine and 33 μm of compound III, ROS formation increased very rapidly (peak in about 30 min, curve not shown) (Table 1). The antioxidants: catalase, superoxide dismutase (SOD), butylatedhydroxytoluene (BHT) and ROS scavengers (26) mannitol and dimethylsulfoxide (DMSO) and the ferric chelator (desferoxamine) protected the hepatocytes against both DTIC and compound III induced cytotoxicity as well as ROS generation (Table 1). All of these agents did not show any toxic effect on hepatocytes at the concentrations used (data not shown). However, the CYP2E1 inhibitor phenylimidazole (26-30) and P_450_ reductase inhibitor diphenyliodonium chloride (DPI) (26-30) showed significant effect on both DTIC and compound III induced cell lysis and ROS formation and protected the hepatocytes against dacarbazine and compound IIItoxicity (Table 1). Endocytosis inhibitors including lysosomotropic agents (chloroquine (31) and methylamine (32)) also protected the hepatocytes against DTIC and compound III induced cell lysis and ROS formation (Table1). All of these agents did not show any toxic effect on hepatocytes at the concentrations used (data not shown). Cytotoxicity and ROS generationwere prevented by mitochondrial MPT pore sealing agents (carnitine and cyclosporine) (Table1).

**Table 1:** Effect of antioxidant, ROS scavengers, ferric chelator, MPT pore sealing agents, lysosomotropic agents, and P_450_ reductase inhibitor on DTIC and Compound III -induced hepatocyte cytotoxicity and ROS formation

**Addition**	**Cytotoxicity % (3h)**	**ROS (30min)**
None	20± 2	79 ± 4
Dacarbazine (56 μM )	76 ± 4(1)	230 ± 4(1)
+Catalase (200 U/mL)	46 ± 2(2)	116 ± 5(2)
+Superoxide dismutase (100 U/mL)	45 ± 3(2)	122 ± 2(2)
+BHT (50 μM)	42 ± 3(2)	118 ± 4(2)
+Mannitol (50 mM)	48 ± 3(2)	136 ± 3(2)
+Dimethyl sulfoxide (150 μM)	44 ± 3(2)	121 ± 2(2)
+Phenylimidazole (300 μM)	52 ± 3(2)	161 ± 3(2)
+Diphenyliodoniumchloride (50 μM)	48 ± 5(2)	166 ± 3(2)
+Methylamine (30 mM)	36 ± 4(2)	117 ± 3(2)
+Chloroquine (100 μM)	40 ± 3(2)	128 ± 2(2)
+Desferoxamine (200 μM)	36 ± 2(2)	121 ± 3(2)
+Cyclosporine (2 μM)	34 ± 3(2)	138 ± 3(2)
+Carnitine (2 mM)	37 ± 4(2)	152 ± 3(2)
**Compound III (33 **μ**M)**	73 ± 2(1)	256 ± 5(1)
+Catalase (200 U/mL)	38 ± 2(3)	126 ± 3(3)
+Superoxide dismutase (100 U/mL)	41 ± 4(3)	132 ± 2(3)
+BHT(50 μM)	37 ± 4(3)	128 ± 2(3)
+Mannitol (50 mM)	38 ± 4(3)	141 ± 3(3)
+Dimethyl sulfoxide (150 μM)	36 ± 3(3)	145 ± 2(3)
+Phenylimidazole (300 μM)	48 ± 5(3)	162 ± 3(3)
+Diphenyliodoniumchloride (50 μM)	48 ± 5(3)	167 ± 4(3)
+Methylamine (30 mM)	31 ± 2(3)	141 ± 2(3)
+Chloroquine (100 μM)	46 ± 3(3)	155 ± 3(3)
+Desferoxamine (200 μM)	35 ± 3(3)	136 ± 3(3)
+Cyclosporine (2 μM)	28 ± 2(3)	141 ± 2(3)
+Carnitine (2 mM)	31 ± 3(3)	161 ± 3(3)

Hepatocytes (106 cells/mL) were incubated in Krebs-Henseleit buffer pH 7.4 at 37 ˚C for 3 h following the addition of DTIC and Compound III. Cytotoxicity was determined as the percentage of cells that take up trypan blue (19, 33). DCF formation was expressed as fluorescent intensity units (34). Values are expressed as means of three separate experiments (SD).

(1) Significant difference in comparison with control hepatocytes (p < 0.05).

(2) Significant difference in comparison with DTIC treated hepatocytes (p < 0.05).

(3) Significant difference in comparison with compound III treated hepatocytes (p < 0.05).

When hepatocyte lysosomes were preloaded with acridine orange, release of acridine orange into the cytosolic fraction ensued within 30min after treating the loaded hepatocytes with 56 μM of DTIC and 33 μm of compound III (Table 2). The DTIC- and compound III-induced acridine orange release, which is a marker of lysosomal membrane damage, was prevented by ROS scavengers including dimethylsulfoxide, mannitol and antioxidants such as catalase,butylatedhydroxytoluene (BHT), superoxide dismutase (SOD) or the ferric chelatordesferoxamine (Table2). Phenylimidazole and diphenyliodonium chloride (DPI) also inhibited dacarbazine and compound III acridine orange release (Table 2). Dacarbazine- and compound III- induced acridine orange redistribution was prevented by chloroquine and methylamine (Table 2). None of these agents alone at the concentrations used showedany significant effect on acridine orange release in acridine orange-loaded hepatocytes (data not shown).

**Table 2:** Preventing DTIC and Compound III induced hepatocyte lysosomal membrane damage by antioxidants, ROS scavengers, ferric chelator, CYP2E1 inhibitor, P_450_ reductase inhibitor, lysosomotropic agents

**Addition**	**% Acridine orange redistribution** **Incubation Time**		
	**2 min**	**15 min**	**30 min**
None	2 ± 1	4 ± 2	4 ± 3
Dacarbazine (56 μM )	183 ± 5(1)	237 ± 5(1)	250 ± 4(1)
+Catalase (200 U/mL)	11 ± 1(2)	14 ± 2(2)	18 ± 2(2)
+SOD (100 U/mL)	10 ± 2(2)	16 ± 2(2)	20 ± 2(2)
+BHT (50 μM)	14 ± 1(2)	19 ± 2(2)	25 ± 3(2)
+Mannitol (50 mM)	8 ± 2(2)	11 ± 1(2)	13 ± 1(2)
+Dimethyl sulfoxide (150 μM)	8 ± 3(2)	10 ± 1(2)	12 ± 1(2)
+Phenylimidazole (300 μM)	16 ± 2(2)	22 ± 1(2)	30 ± 3(2)
+Diphenyliodoniumchloride (50 μM)	18 ± 3(2)	26 ± 3(2)	33 ± 3(2)
+Methylamine (30 mM)	8 ± 2(2)	11 ± 1(2)	14 ± 1(2)
+Chloroquine (100 μM)	12 ± 1(2)	15 ± 2(2)	20 ± 2(2)
+Desferoxamine (200 μM)	8 ± 2(2)	10 ± 2(2)	11 ± 1(2)
Compound III (33 μM)	194 ± 5(1)	240 ± 5(1)	264 ± 5(1)
+Catalase (200 U/mL)	12 ± 1(3)	16 ± 2(3)	18 ± 2(3)
+SOD (100 U/mL)	10 ± 2(3)	15 ± 2(3)	21 ± 2(3)
+BHT (50 μM)	16 ± 2(3)	20 ± 3(3)	24 ± 3(3)
+Mannitol (50 mM)	12 ± 1(3)	16 ± 1(3)	19 ± 2(3)
+Dimethyl sulfoxide (150 μM)	14 ± 2(3)	18 ± 2(3)	21 ± 2(3)
+Phenylimidazole (300 μM)	15 ± 2(3)	17 ± 2(3)	24 ± 2(3)
+Diphenyliodoniumchloride (50 μM)	18 ± 3(3)	22 ± 2(3)	28 ± 3(3)
+Methylamine (30 mM)	12 ± 1(3)	14 ± 1(3)	17 ± 2(3)
+Chloroquine (100 μM)	16 ± 2(3)	18 ± 2(3)	25 ± 2(3)
+Desferoxamine (200 μM)	10 ± 1(3)	12 ± 1(3)	16 ± 2(3)

Hepatocytes (106 cells/mL) were incubated in Krebs-Henseleit buffer pH 7.4 at 37 ˚C for 30 min. Lysosomal membrane damage was determined as intensity unit of diffuse cytosolic green fluorescence induced by acridine orange following the release from lysosome (16).

Values are expressed as means of three separate experiments (SD).

(1) Significant difference in comparison with control hepatocytes (p < 0.05).

(2) Significant difference in comparison with DTIC treated hepatocytes (p < 0.05).

(3) Significant difference in comparison with compound III treated hepatocytes (p < 0.05).

As shown in Table 3, DTIC(56 μM) and Compound III(33 μM) induced a rapid decline of mitochondrial membrane potential (42% and 55% respectively) immediately after the incubation with hepatocytes and 77% and 90% after 30min of incubation at 37 °C which was prevented by reactive oxygen species scavengers (mannitol, DMSO), antioxidants (butylatedhydroxytoluene, catalase), suggesting that the observed decrease in mitochondrial membrane potential which was induced by DTIC and Compound III, was due to reactiveoxygen species formation. In addition, the NADPH P_450_ reductaseinhibitor, diphenyliodonium chloride and reduced CYP2E1 inhibitor, phenylimidazole, inhibited the decline of mitochondrialmembrane potential. Mitochondrial membrane potential collapse was prevented by mitochondrial MPT pore sealing agents (carnitine and cyclosporine) (Table 3). All of these reagents including radicalscavengers, antioxidants, MPT pore sealing agents, NADPH P_450_ reductase inhibitor and reduced CYP2E1 inhibitor did not show any significant effect on hepatocyte mitochondrial membrane potential at the concentrations used while incubated alone (data not shown).

**Table 3:** Mitochondrial membrane potential changes during DTIC and Compound III induced hepatocyte injury by antioxidants, ROS scavengers, CYP2E1 inhibitor, P450 reductase inhibitor and mitochondrial MPT pore sealing agents

**ΔΨm%Incubation Time**			Addition	
min 30	min 15	min 2		
4 ± 2	3 ± 1	2 ± 1		None
77 ± 3(1)	56 ± 2(1)	42 ± 3(1)		**Dacarbazine (56 **μ**M )**
16 ± 2(2)	10 ± 3(2)	6 ± 2(2)		+Catalase (200 U/mL)
20 ± 3(2)	14 ± 2(2)	6 ± 3(2)		+BHT(50 μM)
21 ± 2(2)	16 ± 2(2)	9 ± 3(2)		+Mannitol (50 mM)
18 ± 2(2)	14 ± 3(2)	6 ± 2(2)		+Dimethyl sulfoxide (150 μM)
15 ± 1(2)	9 ± 3(2)	6 ± 3(2)		+Phenylimidazole (300 μM)
19 ± 2(2)	12 ± 1(2)	8 ± 2(2)		+Diphenyliodoniumchloride (50 μM)
16 ± 2(2)	10 ± 1(2)	8 ± 3(2)		+Cyclosporine (2 μM)
19 ± 2(2)	12 ± 2(2)	8 ± 2(2)		+Carnitine (2 mM)
90 ± 4(1)	66 ± 6(1)	55 ± 1(1)		**Compound III(33 **μ**M)**
18 ± 2(3)	12 ± 2(3)	7 ± 2(3)		+Catalase (200 U/mL)
18 ± 3(3)	15 ± 3(3)	10 ± 3(3)		+BHT(50 μM)
22 ± 2(3)	17 ± 2(3)	9 ± 2(3)		+Mannitol (50 mM)
18 ± 2(3)	15 ± 2(3)	8 ± 3(3)		+Dimethyl sulfoxide (150 μM)
22 ± 2(3)	16 ± 3(3)	10 ± 1(3)		+Phenylimidazole (300 μM)
26 ± 3(3)	18 ± 2(3)	11 ± 1(3)		+Diphenyliodoniumchloride (50 μM)
16 ± 2(3)	11 ± 3(3)	5 ± 2(3)		+Cyclosporine (2 μM)
20 ± 2(3)	14 ± 2(3)	10 ± 1(3)		+Carnitine (2 mM)

Hepatocytes (106 cells/mL) were incubated in Krebs-Henseleit buffer pH 7.4 at 37 ˚C for 30 min. Mitochondrial membrane potential was determined as the difference in mitochondrial uptake of the rhodamine 123 between control and treated cells. Our data were shown as the percentage of mitochondrial membrane potential collapse (%ΔΨm) in all treated (test) hepatocyte groups (22, 35).

Values are expressed as means of three separate experiments (SD).

(1) Significant difference in comparison with control hepatocytes (p < 0.05).

(2) Significant difference in comparison with DTIC treated hepatocytes (p < 0.05).

(3) Significant difference in comparison with compound III treated hepatocytes (p < 0.05).

As shown in Table 4, DTIC and Compound III induced caspase-3 activity in hepatocytes. Antioxidants (butylatedhydroxytoluene, catal ase), reactive oxygen species scavengers (mannitol, DMSO), NADPH P_450_ reductase inhibitor (diphenyliodonium chloride) and reduced CYP2E1 inhibitor (phenylimidazole), significantly decreased the activity of caspase-3. Mitochondrial MPT pore sealing agents (carnitine and cyclosporine) significantly decreased the activity of caspase-3 in comparison with hepatocytes incubated by DTIC and Compound III (Table 4).

**Table 4:** Blockade ofDTIC and Compound III induced Caspase-3 activation by antioxidants, ROS scavengers, CYP2E1 inhibitor, p_450_ reductase inhibitor and mitochondrial MPT pore sealing agents

**Addition**	**Caspase-3 Activity** **2 h**
None	297.65 ± 5
Dacarbazine (56 μM )	659.52 ± 7(1)
+Catalase (200 U/mL)	258.74 ± 5(2)
+BHT (50 μM)	260.52 ± 3(2)
+Mannitol (50 mM)	285.46 ± 4(2)
+Dimethyl sulfoxide (150 μM)	281.33 ± 5(2)
+Phenylimidazole (300 μM)	223.39 ± 3(2)
+Diphenyliodoniumchloride (50 μM)	244.65 ± 4(2)
+Cyclosporine (2 μM)	116.32 ± 2(2)
+Carnitine (2 mM)	124.38 ± 3(2)
Compound III(33 μM)	634.78 ± 5(1)
+Catalase (200 U/mL)	138.73 ± 4(3)
+BHT (50 μM)	149.13 ± 3(3)
+Mannitol (50 mM)	281.22 ± 3(3)
+Dimethyl sulfoxide (150 μM)	251.18 ± 2(3)
+Phenylimidazole (300 μM)	173.89 ± 3(3)
+Diphenyliodoniumchloride (50 μM)	188.36 ± 3(3)
+Cyclosporine (2 μM)	96.18 ± 2(3)
+Carnitine (2 mM)	99.63 ± 2(3)

Hepatocytes (106 cells/mL) were incubated in Krebs-Henseleit buffer pH 7.4 at 37 ˚C for 30 min. The activating of caspase-3 (μM pNA/min/mL)) was determined based on hydrolysis of pNA labeled substrate (36). Values are expressed as means of three separate experiments (SD)

(1) Significant difference in comparison with control hepatocytes (p < 0.05).

(2) Significant difference in comparison with DTIC treated hepatocytes (p < 0.05).

(3) Significant difference in comparison with compound III treated hepatocytes (p < 0.05).

## Conclusion

The preliminarystudies for determination of EC_50_ of dacarbazine andcompound IIIreveals that the cytotoxicity of compound III is comparable with that of dacarbazine.Comparison ofthe EC_50 _values for the two compounds even shows higher potency for compound III.

The results of cellular-molecular mechanistic studies for compound III and dacarbazine indicate that the two compounds show the same pattern of cytotoxicityform the mechanistic point of view.

In light of the fact that in compound III, an un-substituted pyridine ring hadreplaced the carbamoyl-imidazole ring in dacarbazine, it could be concluded that the imidazole ring and its dynamic tautomerization do not have significant role in dacarbazineinduced cytotoxic activity. Complementary detailed mechanistic studies such as investigation ofthe DNA-methylating properties for compound III will be further helpful to verify the accuracy of the above suggestion.
